# Polygenic Risk Score: Clinically Useful Tool for Prediction of Cardiovascular Disease and Benefit from Lipid-Lowering Therapy?

**DOI:** 10.1007/s10557-020-07105-7

**Published:** 2020-11-06

**Authors:** Natalie Arnold, Wolfgang Koenig

**Affiliations:** 1 Department of Cardiology, University Heart and Vascular Center Hamburg, Hamburg, Germany; 2 German Center for Cardiovascular Research (DZHK), partner site Hamburg/Kiel/Luebeck, Hamburg, Germany; 3 Deutsches Herzzentrum München, Technische Universität München, Lazarettstr. 36, 80636 Munich, Germany; 4 German Centre for Cardiovascular Research (DZHK), partner site Munich Heart Alliance, Munich, Germany; 5 Institute of Epidemiology and Medical Biometry, University of Ulm, Ulm, Germany

**Keywords:** Genetic risk scores, Prediction, Cardiovascular disease, Response to treatment, Precision medicine

## Abstract

Improvement in risk prediction of atherosclerotic cardiovascular disease (ASCVD) using information on the genetic predisposition at an individual level might offer new possibilities for the successful management of such complex trait. Latest developments in genetic research with the conduction of genome-wide association studies have facilitated a broader utility of polygenic risk score (PRS) as a potent risk prognosticator, being strongly associated with future cardiovascular events. Although its discriminative ability beyond traditional risk factors is still a matter of controversy, PRS possesses at least comparable risk information to that provided by traditional risk tools. More importantly, increased genetic risk for ASCVD might be discovered at younger ages, much longer before conventional risk factors become manifest, thereby providing a potent instrument for aggressive primordial and primary prevention in those at high risk. Furthermore, there is strong evidence that inherited risk may be successfully modulated by a healthy lifestyle or medication use (e.g., statins or PCSK-9 inhibitors). Here, we provide a short overview of the current research related to the possible application of PRS in clinical routine and critically discuss existing pitfalls, which still limit a widespread utility of PRS outside a research setting.

## Current Gaps in Risk Prediction of Atherosclerotic Cardiovascular Disease—Scope of the Problem

Prediction of atherosclerotic cardiovascular disease (ASCVD), its first or recurrent events, remains an ongoing challenge. Despite the widespread clinical application of various risk assessment tools [1, 2], there is still a large number of subjects at risk, who remain unidentified until their first event (Rose Paradoxon) [3]. All risk calculators, currently used in the primary prevention setting, are based on generally available clinical characteristics, mainly traditional risk factors. However, recently published data from the Progression of Early Subclinical Atherosclerosis (PESA) study showed that even in the absence of conventional risk factors a large proportion of subjects (up to 50% out of ~ 4200 study participants) already had subclinical atherosclerosis as assessed by noninvasive imaging (carotid ultrasound or coronary artery calcium) [4]. Moreover, most of the subjects were also on “optimal” treatment regarding traditional risk factors (blood pressure < 120/80 mmHg, fasting glucose < 100 mg/dl, hemoglobin A1c < 5.7%, and total cholesterol < 200 mg/dl), thereby having “successfully achieved” preventive measures. A similar situation can be observed in patients with manifest atherosclerotic disease, where more than 20% of patients with established coronary heart disease (CHD) experience a recurrent event within 5 years after an index event, despite significantly improved aggressive treatment strategies including early mechanical intervention and polypharmacotherapy [5, 6]. Thus, despite major efforts, still a significant number of future ASCVD events (incident or recurrent) cannot be explained by traditional risk factors only.

This scenario has prompted the search for other predictors of increased cardiovascular risk, e.g., novel blood biomarkers related to the pathophysiology of ASCVD. Although initial views were very enthusiastic that circulating proteins might convey the potential for improved risk stratification, currently, only a small number of biomarkers (mainly high sensitive (hs) troponins, natriuretic peptides, or hs C-reactive protein (CRP)) have been successfully introduced in the clinical routine, despite all efforts and years of extensive investigations. Even for proteomics, a novel promising technology to detect, profile, and quantify protein biomarkers, data are controversial regarding the clinically significant improvement of cardiovascular risk assessment, although its crucial role in better understanding the etiology and pathophysiology of CVD should not be underestimated [7].

The challenge that exists in risk prediction of such complex trait like ASCVD is also partially caused by its substantial heritability (up to 60%) and its polygenic architecture [8]. Therefore, a considerable scientific interest on how genetic factors contribute to the development of ASCVD or whether genetic information (as a single-gene variation or as an overall genetic burden) might be used to specify prediction of outcome is not surprising. Over the past decade, the development of large genotyping arrays together with concomitant advances in the statistical methodologies has resulted in a considerable progress within the field of genomics research with conduction of several genome-wide association studies (GWASs) [9–11]. Since 2007, GWASs have uncovered more than 150 common genetic loci (so-called single-nucleotide polymorphisms (SNPs)) that are robustly associated with ASCVD or CHD in particular [12]. Several of them are related to alterations in conventional risk factors (lipoprotein metabolism, insulin resistance) or other important pathophysiological pathways (thrombosis, inflammation, or regulation of the vascular tone), whereas the mechanisms of action of numerous novel genetic regions such as e.g. for the most impactful CHD-related locus 9p21.3 [9–11] are still not entirely elucidated, although several potential mechanisms have been proposed [13].

## From Single-Nucleotide Polymorphisms to Polygenic Scores

Although most of the discovered genetic variants are most likely causally involved in the pathogenesis of ASCVD (e.g., IL-6, as proven by Mendelian Randomization studies [14]), if viewed individually, single SNPs explain only a small proportion of increased risk conferred by the genetic background and hence cannot be reliably used in disease prediction. Thus, recent scientific activities have focused on the assessment of aggregate information from single SNPs (each associated with the outcome of interest but with only very small true effect size) with subsequent building of a genetic risk score (also called a polygenic risk score (PRS)). Polygenic risk scores represent a summary effect of all the risk variants for a trait in an individual and can be considered as a quantitative measure of genetic susceptibility. To date, there are multiple approaches and rules to construct PRSs, which are however beyond the scope of this review (for more information, see ref. [15–17]). In general, the PRS might include a small set of highly significant SNPs, which passed genome-wide significance thresholds or be conducted from millions of “low-impact” SNPs with only modest contribution to the phenotype of interest.

One of the earliest studies evaluating the prognostic ability of PRS for incident ASCVD was performed in 2008 by Kathiresan et al. [18]. Using data from 5414 subjects from the cardiovascular cohort of the Malmo Diet and Cancer Study (MDCS), the authors constructed an unweighted GWAS-based PRS comprising 9 SNPs involved in low-density lipoprotein (LDL)/high-density lipoprotein (HDL) cholesterol (C) regulation and found them to be predictive for incident ASCVD (i.e., myocardial infarction, ischemic stroke, and death from CHD) over a median follow-up of 10 years. Subjects with a higher genetic risk score demonstrated a 63% increased risk for future events compared to those with a low PRS, even after adjustment for baseline lipid levels and a full set of conventional cardiovascular risk factors. Although in the total population the C statistics for cardiovascular events did not differ between the risk models with or without the genetic score, a modest improvement in risk classification by adding genetic data was found in subjects of intermediate risk (approximately 9% of the study sample), among whom 26% were reclassified into a higher or lower risk category. Almost identical results were obtained within the same study with regard to the CHD-specific PRS, including 13 SNPs from CHD risk loci, identified by initial GWAS [19]. Again, a weighted PRS was strongly associated with incident CHD with a hazard ratio (HR) of 1.66 comparing extreme quintiles of the PRS after multivariable adjustment for clinical covariates. Since then, a series of well-conducted studies have been published, providing strong evidence for a robust association between increased genetic risk with prevalent or incident ASCVD [18–30] (see Table 1).

**Table 1:** Genetic risk and atherosclerotic cardiovascular disease: overview of published studies

Authors	Year	Study	Outcome	Risk estimate	Ref.
Kathiresan et al.	2008	MDCS	Incident ASCVD	HR 1.15 (95% CI 1.07–1.24) per copy of an unfavorable allele	[18]
Rapatti et al.	2010	MDCS	Incident CHD	HR 1.66 (95% CI 1.35–2.04) for high vs low genetic risk	[19]
Mega et al.	2015	MDCS, JUPITER, ASCOT (combined)	Incident CHD	HR 1.72 (95% CI 1.53–1.92) for high vs low genetic risk	[20]
		CARE, PROVE IT (combined)	Recurrent CHD	HR 1.81 (95% CI 1.22–2.67) for high vs low genetic risk	
de Vries et al.	2015	Rotterdam	Incident CHD	HR 1.13 (95% CI 1.06–1.20) per 1 SD increase in PRS	[21]
Khera et al.	2016	ARIC, WGHS, MDCS (combined)	Incident CHD	HR 1.91 (95% CI 1.75–2.09) for high vs low genetic risk	[22]
Tada et al.	2016	MDCS	Incident CHD	HR 1.92 (95% CI 1.67–2.20) for high vs low genetic risk	[23]
Natarajan et al.	2017	WOSCOPS	Incident CHD	HR 1.66 (95% CI 1.21–2.29) for high vs low genetic risk	[24]
Inoya et al.	2018	UK Biobank	Prevalent and Incident CHD	HR 1.71 (95% CI 1.68–1.73) per 1 SD increase in PRS	[25]
Wünnemann et al.	2019	MHI Biobank, CARTaGENE (combined)	Prevalent CHD	OR 1.69 (95% CI 1.58–1.81) per 1 SD increase in PRS	[26]
Mostley et al.	2020	ARIC	Incident CHD	HR 1.24 (95% CI 1.15–1.34) per 1 SD increase in PRS	[27]
		MESA	Incident CHD	HR 1.38 (95% CI 1.21–1.58) per 1 SD increase in PRS	
Elliott et al.	2020	UK Biobank	Incident CHD	HR 1.32 (95% CI 1.30–1.34) per 1 SD increase in PRS	[28]
Marston et al.	2020	FOURIER	MACE	HR 1.65 (95% CI 1.30–2.10) for high vs low genetic risk	[29]
Damask et al.	2020	ODYSSEY-OUTCOMES	MACE	HR 1.59 (95% CI 1.28–1.96) for high vs low genetic risk	[30]

*MDCS*, Malmo Diet and Cancer Study; *ASCVD*, atherosclerotic cardiovascular disease; *HR*, Hazard ratio; *CI*, confidence interval; *JUPITER*, Justification for the Use of Statins in Prevention: an Intervention Trial Evaluating Rosuvastatin; *ASCOT*, Anglo-Scandinavian Cardiac Outcomes Trial; *CARE*, Cholesterol and Recurrent Events; *PROVE IT TIMI 22*, Pravastatin or Atorvastatin Evaluation and Infection Therapy - Thrombolysis in Myocardial Infarction 22; *PRS*, polygenic risk score; *ARIC*, Atherosclerosis Risk in Communities; *WGHS*, Women’s Genome Health Study; *WOSCOPS*, West of Scotland Coronary Prevention Study; *SD*, standard deviation; *MHI*, Montreal Heart Institute; *OR*, odds ratio; *FOURIER*, Further Cardiovascular Outcomes Research With PCSK9 Inhibition in Subjects With Elevated Risk; *MACE*, major advanced coronary events; *ODYSSEY-OUTCOMES*, Evaluation of Cardiovascular Outcomes After and Acute Coronary Syndrome During Treatment with Alirocumab

Based on the aforementioned extensive genetic research during recent years, it has become clear that an unfavorable genetic background, expressed by a high genetic risk score, is predictive for incident and recurrent ASCVD events. In contrast, data on the clinical utility of PRS beyond traditional CVD risk factors or other clinical estimates yielded rather mixed results. Although only modest and probably clinically marginal improvements in risk reclassification by PRS beyond traditional risk factors were seen in several earlier studies, which mostly utilized genetic variants with genome-wide significance or even preselected loci [18, 19, 22, 23], Inoya et al. [25] among > 480,000 individuals from the UK Biobank were able to show that a 1.7 million SNPs PRS had a higher discriminative capacity for incident CHD than any of 6 conventional risk factors such as smoking, diabetes, hypertension, body mass index, self-reported high cholesterol, or family history. Furthermore, Mostley et al. [27] using data from 7306 participants of European ancestry from two population-based cohorts (Atherosclerosis Risk in Communities (ARIC) Study and the Multi-Ethnic Study of Atherosclerosis (MESA)) compared the predictive ability of PRS with and in addition to a guideline-recommended clinical risk equation for a 10-year first CHD event. Despite a strong association of PRS with 10-year CHD incidence (Table 1), no significant change in the C statistics was observed if genetic information was added to the pooled cohort equations (PCE) in the ARIC Study (λC − 0.001), and only a minimal increase was seen among MESA participants (λC 0.021). Furthermore, the addition of the PRS to the PCE did not improve the classification accuracy in a significant way (net reclassification improvement (NRI) 0.018 in ARIC and 0.001 in MESA). It should, however, be noted here that when evaluated separately, the PRS and PCE demonstrated comparable C statistics in both cohorts (PRS/age/sex versus PCE (including age and sex) in ARIC 0.669 versus 0.701; in MESA 0.672 versus 0.660 respectively) [27]. Similarly, among 352,660 participants of the UK Biobank cohort, no differences in C statistics for PRS were seen in a model that included age and sex (C statistics 0.76) versus PCE alone (C statistics 0.76) [28]. However, the combination of a genetic tool with a clinical risk score was associated with a modest but statistically significant improvement in the discriminative accuracy for incident CHD, compared with PCE alone (λC 0.02). Finally, preliminary data from two prospective cohort studies—MDCS (*n* = 5660, 812 incident CAD, median FU 23.2 years) and UK Biobank (*n* = 280,265 individuals with 6727 events, median FU 8.1 years)—further confirmed the abovementioned results revealing no differences in C statistics [31]. However, novel intriguing findings from these analyses showed that within each PCE category PRS added incremental information with markedly (2- to 4-fold) higher rates of incident CHD among those at high (top 20% of PRS) versus low polygenic risk (bottom 20% of PRS).

All the above discussed studies emphasize the fact that PRS is not only a strong predictor of ASCVD, but it also possesses at least comparable risk information than that given by standard risk factors, thereby making adequate risk assessment possible even before a pathologic risk profile has developed. That places an unfavorable genetic background as a key element not only in primary or secondary but probably also in primordial disease prevention.

## Healthy Lifestyle Can at Least Partially Compensate for High Genetic Risk

**Fig. 1:**
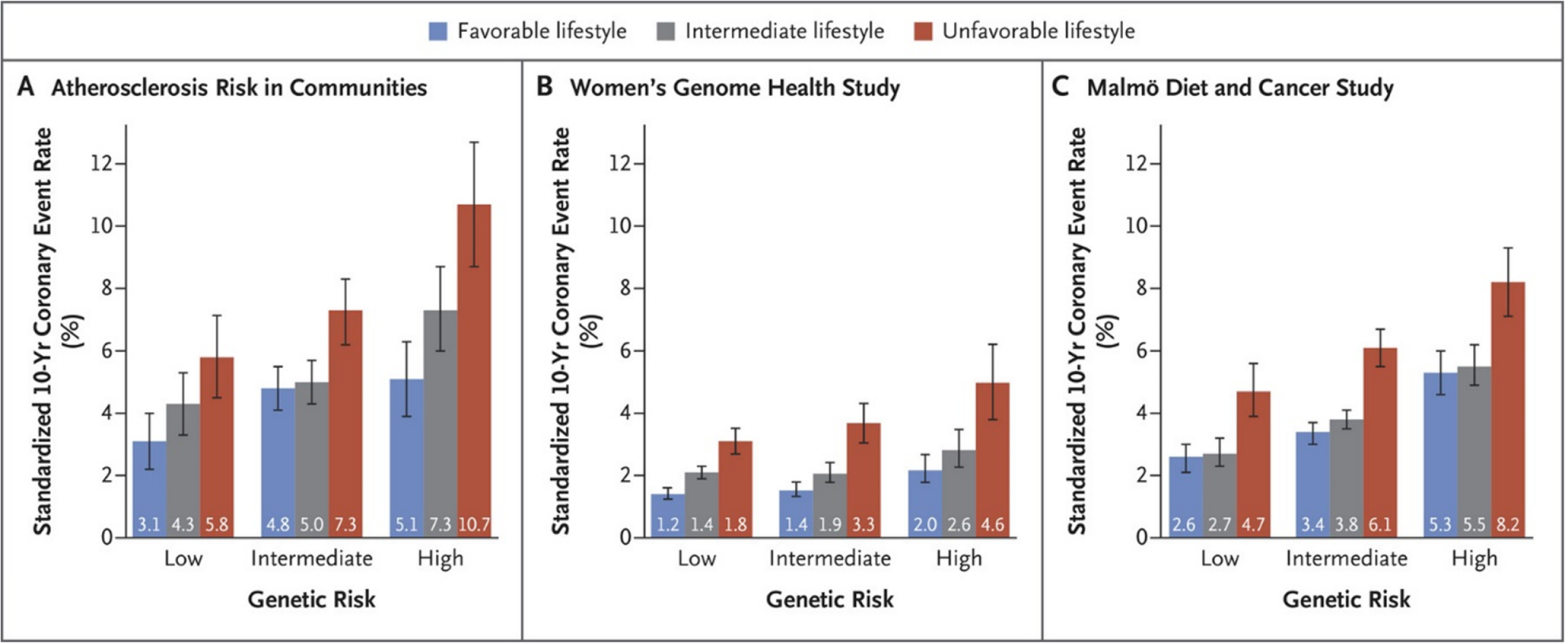
Ten-year coronary event rates, according to lifestyle and genetic risk in the prospective cohorts. Shown are standardized 10-year cumulative incidence rates for coronary events in the three prospective cohorts, according to lifestyle and genetic risk. Standardization was performed to cohort-specific population averages for each covariate. The I bars represent 95% confidence intervals. Reproduced with permission from Khera et al. [22]. Copyright Massachusetts Medical Society. License Number 4879260716155

The question that remains to be answered is whether subjects with increased genetic risk might benefit from early interventional strategies (lifestyle changes or pharmacological treatment). In general, PRS can be considered as a “baseline” genetic risk, which is stable from birth, but can it be modulated externally during lifetime. Indeed, post hoc analyses of numerous clinical trials have demonstrated that high genetic risk for ASCVD might be mitigated by statin use and healthy lifestyle. For instance, across four studies involving 55,685 participants, Khera et al. [22] showed that subjects with high genetic risk but favorable lifestyle have an almost identically increased risk for incident CHD as compared to subjects with low genetic risk but unfavorable lifestyle risk (HR 1.90 (95% CI 1.62–2.23) versus 1.82 (95% CI 1.51–2.19), respectively). Furthermore, among participants with high genetic risk, healthy lifestyles (defined as at least three of the four healthy lifestyle factors) lowered ASCVD event rates by almost 50% (compared with rates for unhealthy lifestyles), thereby suggesting that at least part of the inherited cardiovascular risk can also be compensated by a healthy lifestyle (Fig. 1).

## Genetic Risk Scores to Identify Those with Most Pronounced Benefit from Statin Treatment

Other potential implications of a genetic risk score in clinical practice might relate to the identification of a group of patients who would benefit most from pharmacological therapy. Interesting findings come from post hoc analysis of four statin trials of both primary (Justification for the Use of Statins in Prevention: an Intervention Trial Evaluating Rosuvastatin (JUPITER) and Anglo-Scandinavian Cardiac Outcomes Trial (ASCOT)) and secondary prevention (Cholesterol and Recurrent Events (CARE) and Pravastatin or Atorvastatin Evaluation and Infection Therapy - Thrombolysis in Myocardial Infarction 22 (PROVE IT-TIMI 22)) [20], where individuals with the highest genetic scores had the greatest benefit from statin therapy. A differential gradient in relative risk reduction (RRR) should be noted here, since an almost 50% RRR was seen in subjects at high genetic risk in both primary and secondary prevention trials, as compared to placebo, whereas in those with low genetic risk, the RRR was found to be only 3% in CARE and PROVE IT-TIMI and 34% in the primary prevention trials. Also, the number needed to treat (NNT) over 10 years to prevent one event differed significantly by genetic risk being NNT = 25 in the JUPITER trial and NNT = 20 in ASCOT in those with a high genetic score and an almost three times higher NNT in participants with a low genetic risk (NNT = 66 in JUPITER and NNT = 57 in ASCOT). These results were further corroborated within another randomized controlled statin trial in primary prevention (West of Scotland Coronary Prevention Study (WOSCOPS); *n* = 4910) [24], where a similar RRR of 44% was found among the high PRS group on statin therapy.

## Genetic Risk Scores to Identify Those with the Greatest Benefit from Treatment with PCSK9 Inhibitors—a Precision Medicine and Pharmacoeconomic Approach

**Fig. 2:**
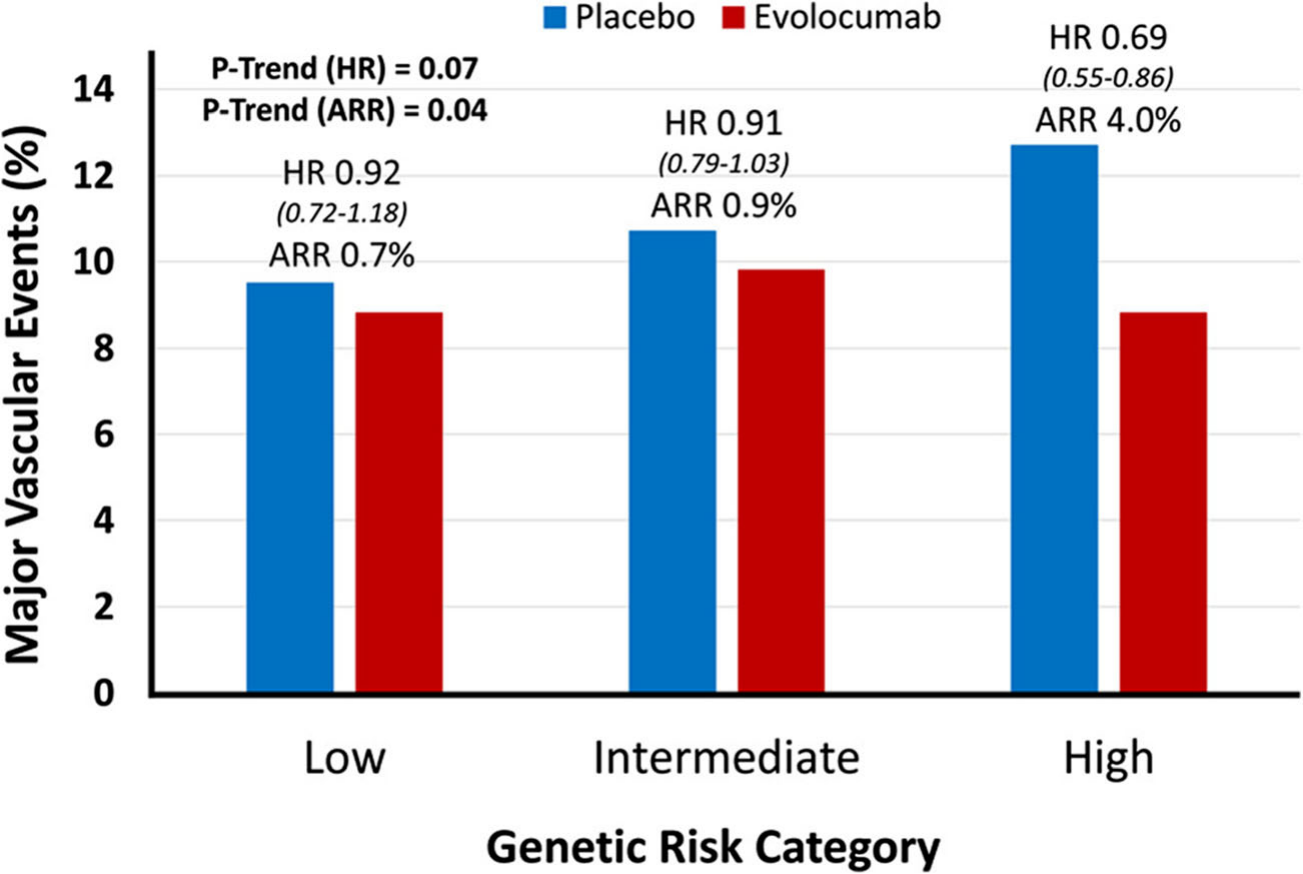
Relative and absolute risk reduction of major vascular events with evolocumab, by genetic risk category: results from the FOURIER trial. ARR indicates absolute risk reduction, and HR, hazard ratio. Reproduced with permission from Marston et al. [29]. Copyright Wolters Kluwer Health. License Number 4879241288878

Thus, information on the genetic background might provide useful information in the selection of subjects who can clearly benefit from early treatment initiation. That might be of particular importance if the widespread use of a drug treatment is limited by its high costs. Prominent examples for such strategy have recently been published from the FOURIER and ODYSSEY trials [29, 30], both evaluating compounds derived from a new class of very effective LDL-C lowering drugs, proprotein convertase subtilisin/kexin type 9 (PCSK9) inhibitors evolocumab and alirocumab in patients with established ASCVD. Within FOURIER [29], a multicenter, randomized, double-blind, placebo-controlled trial on evolocumab to reduce major adverse cardiovascular events (MACE) in statin-treated patients with ASCVD, a 27 SNP risk score has been applied among 14,298 enrolled participants. Surprisingly, subjects within the high genetic risk group rather had a more favorable risk profile including a lower prevalence of smoking and diabetes mellitus, yet increasing age or male sex compared to the individuals in the low- or intermediate-risk group, thereby again highlighting a captured residual risk left unaccounted by traditional cardiovascular risk factors. Furthermore, a comparable baseline concentration of LDL-C has been observed between all three subgroups (94 mg/dl in the patients with high risk versus 92 mg/dl in the patients with intermediate risk, or 91 mg/dl in the patients with low genetic risk). However, the most intriguing finding from this analysis was related to the fact that only subjects with a high genetic risk received the greatest absolute and relative benefit from PCSK9 inhibition. Being in the high genetic risk category was associated with a pronounced risk reduction of major vascular events (HR 0.69 (95% CI 0.55–0.86)), which was quite stronger than in the overall population with a 15% RRR. In fact, patients with a low and intermediate genetic risk demonstrated only negligible treatment-associated effects (HR 0.92 (95% CI 0.72–1.18) and 0.91 (95% CI 0.79–1.03), respectively). The authors went further and assessed the clinical benefit from PCSK9 inhibition by combining genetic and clinical risk and found that in patients with a low burden of conventional risk factors and low genetic risk, no benefit of treatment with evolocumab was observed over a median of 2.3 years. In contrast, the high genetic risk was associated with a 31% treatment-related risk reduction of major vascular events irrespective of the CVD risk profile (Fig. 2). Treatment with evolocumab completely mitigated the increased risk in the high-genetic-risk category, lowering their event rate to that of the low-genetic-risk category. Similar findings were observed within the Evaluation of Cardiovascular Outcomes After an Acute Coronary Syndrome During Treatment With Alirocumab (ODYSSEY-OUTCOMES) trial [30] where a RRR for MACE by alirocumab treatment was 37% in the high PRS patients versus 13% in the lower PRS patients. More interestingly, those with high LDL-C and high PRS derived the greatest benefit from alirocumab. Again, substantial mitigation of the adverse genetic risk was observed under PCSK-9 inhibition. However, despite such promising findings from FOURIER and ODYSSEY-OUTCOMES trials, these results also raise one crucial issue—what should we do with those on the “opposite to high” side of risk, i.e., subjects with low genetic but relevant clinical risk, who have a clear indication for LDL-C lowering therapy but no reduction in event rates on PCSK-9 inhibition? Would we then lower LDL-C without lowering CVD risk? Should such individuals be chosen for other aggressive treatment strategies, most probably having other (e.g., inflammatory) residual risks? All these points need urgent consideration in the near future. Thus, there is a clear need for more clinical trials using genomic information to validate such findings.

## Critical Consideration and Future Perspectives

Ultimately, to date, we have no reason to question the importance of genetic information in the prediction of future cardiovascular risk. However, the question that still needs to be answered is the intended purpose of genetic risk assessment, since this might influence the design and further validation of PRS: Do we want to assess risk associated with gene variants, most probably causally involved in the development of atherosclerosis and rather reflecting monogeneity of the trait? Or do we hope to catch an overall increased genetic risk at an individual’s level to better manage it later? Certainly, for sufficient risk prediction, the relationships do not need to be causal and in that case a PRS might rather reflect a network of synergistic interactions of various modulatory effects, like e.g. genetic determinants of CRP or natriuretic peptide concentrations, which most probably have no direct/causal effect on atherosclerosis development, but definitely modulate ASCVD risk. Also, traditional risk factors might be a consequence of such interaction, since they are most probably already modified by the genetic background at the time of assessment. That might be also one reason why PRS possesses only a moderate discriminative ability beyond traditional risk factors, since it cannot be excluded completely that genetic variation was already captured by the intermediate phenotype such as e.g. arterial hypertension or dyslipidemia. To be provocative, do we assess genetic information twice within the same prediction models, once by PRS per se, once by inclusion of clinical variables? Interestingly, this assumption might represent a reasonable explanation why various studies [27, 31] consistently demonstrated that PRS possesses similar predictive performance as conventional risk factors, and this fact cannot be simply ignored.

Taking together, all currently available evidence supports the use of PRS, as a measure of overall genetic susceptibility to ASCVD as a promising tool in risk discrimination or targeting therapies. Nonetheless, there are still a number of open points that need to be clarified before its widespread implementation into clinical practice. First, due to the diversity of currently existing PRS, it is not clear which score should be used to obtain maximal prognostic information. Numerous investigations showed a predictive advantage of more comprehensive scores [21, 23], whereas recent data from the FOURIER [29] and ODYSSEY-OUTCOMES trials [30] revealed that a genome-wide PRS of ≈ 6.6 million variants did not provide a better performance in predicting treatment benefit from PCSK-9 inhibitor therapy than a limited (27 or 57 SNPs with genome-wide significance) PRS. Second, it is also not clear which elements or factors drive high genetic risk and how to identify such a core pathway among a huge number of existing SNPs. Third, in addition, standardization in assays and data processing are urgently required. Fourth, only limited data are available on the utility of PRS in more diverse populations based on race, ethnicity, or other minority groups. For instance, Irribaren et al. [32], investigating the role of PRS generated in European ancestry populations for incident CHD among individuals of African, Latino, and East-Asian ancestry, found that the added predictive value of PRS was more prominent in African-American and Latinos than in East Asians. Fifth, most surprisingly, despite the large numbers of studies relating genetic risk to outcome prediction, only several of them reported sex-specific data, since the association between PRS and events might differ significantly between men and women, as it has been shown recently in MESA [33]. This would support a concept of significant sex-specific disparities at a genome level. And the most important issue is related to how to identify a target population where PRS can be applied more efficiently? Is a uniform application of the same PRS among diverse cohorts the right way to assess a polygenic contribution to disease? Should we really use the same score in completely different settings such as e.g. guidance to medical therapy in those with manifest disease or for primordial and primary prevention among e.g. younger people, i.e., in the population which probably might have the most sustainable benefit from aggressive preventive measures if carrying high genetic risk? All such points require additional consideration in further studies (Table 2).

**Table 2:** Pro and cons of genetic risk stratification in ASCVD

Pros	Genetic predisposition remains unchanged throughout life
	Early assessment of genetic risk before development/exposure of traditional and environmental risk factors
	Low cost of direct-to-consumer tests
	Huge potential in estimating lifetime risk trajectories
	Huge potential in improvement of medical decision-making for: Accelerated preventive measures in those with high genetic risk Initiation of cost-effective therapies (e.g., PCSK9 inhibitors) Assessment of non-responders Prediction of adverse drug effects
	Simultaneous use for a wide range of other complex diseases
Cons	Pitfalls in the PRS construction: Unclear “build-up” strategy: genome-wide thresholds vs “relaxing” (based on millions of SNPs) strategy Unclear optimal weighting strategies Weaker evidence in non-European ancestry
	Pitfalls in the PRS interpretations/methodological research: Categorizing versus dichotomizing of PRS Unclear predictive accuracy beyond traditional risk factors
	Unclear outcome phenotype: comprehensive PRS for common ASCVD or outcome-specific PRS (e.g., separately for stroke/CAD/PAD)
	Unclear target populations for genetic risk stratification
	Unclear mechanisms behind increased genetic risk: true causal *loci* vs gene-gene interactions (*epistasis*) and gene-environment interactions
	Unclear cost-effectiveness

*ASCVD*, atherosclerotic cardiovascular disease; *PCSK9*, proprotein convertase subtilisin/kexin type 9; *SNPs*, single-nucleotide polymorphisms; *PRS*, polygenic risk score; *CHD*, coronary heart disease; *PAD*, peripheral artery disease

**Fig. 3:**
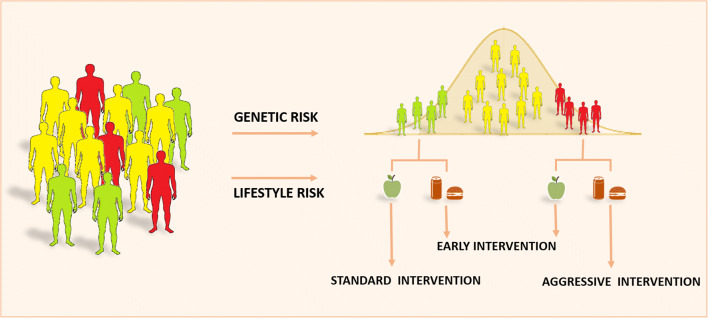
Possible clinical utility of polygenic risk scores. Combined assessment of genetic and lifestyle risk might provide a potent instrument for aggressive primordial and primary prevention

Thus, taken together, the concept of genetic risk shows great promise not only as an early screening tool and decision trigger for accelerated preventive measures, especially in the combination with lifestyle factors (Fig. 3), but also in representing a sound basis for the initiation of cost-effective therapies (e.g., PCSK9 inhibitors), assessment of non-responders, or prediction of adverse drug effects. Most importantly, measured only once during lifetime at relatively low cost (direct-to-consumer tests currently < $100), PRS might be applied for virtually any trait, thereby helping us to provide true precision medicine.

## Conclusion

Information on the genetic background does represent a powerful tool for more personalized clinical assessment and therapy, and we need to put our scientific efforts in overcoming still existing pitfalls related to the limited clinical use of genetic propensity. What is crucial is a clear analytic strategy and central coordination of further research and collaboration within different genetic consortia which might facilitate the translation of such comprehensive research program into real life, hereby providing missing puzzles to justify the widespread application of PRSs outside a research setting. Certainly, genetic predisposition cannot be directly changed, but we now have enough evidence that inherited risk can be successfully mitigated by favorable lifestyle profile and, in certain subjects, even in combination with early pharmacologic interventions e.g. in the lipid metabolism.

## Data Availability

Not applicable.
