# Correction to: Polygenic Risk Score: Clinically Useful Tool for Prediction of Cardiovascular Disease and Benefit from Lipid-Lowering Therapy?

**DOI:** 10.1007/s10557-021-07269-w

**Published:** 2024-09-28

**Authors:** Natalie Arnold, Wolfgang Koenig

**Affiliations:** 1 Department of Cardiology, University Heart and Vascular Center, Hamburg, Germany; 2 German Center for Cardiovascular Research (DZHK), Partner Site, Hamburg/Kiel/Luebeck, Hamburg, Germany; 3 Deutsches Herzzentrum München, Technische Universität München, Lazarettstr. 36, 80636 Munich, Germany; 4 German Centre for Cardiovascular Research (DZHK), Partner Site, Munich Heart Alliance, Munich, Germany; 5 Institute of Epidemiology and Medical Biometry, University of Ulm, Ulm, Germany

**Correction to: Cardiovascular Drugs and Therapy (2021)**

10.1007/s10557-020-07105-7

The article “Polygenic Risk Score: Clinically Useful Tool for Prediction of Cardiovascular Disease and Benefit from Lipid-Lowering Therapy?” written by Natalie Arnold and Wolfgang Koenig, was originally published Online First without Open Access. After publication, the author decided to opt for Open Choice and to make the article an Open Access publication. Therefore, the copyright of the article has been changed to © The Author(s) 2021 and the article is forthwith distributed under the terms of the Creative Commons Attribution 4.0 International License (http://creativecommons.org/licenses/by/4.0/), which permits use, duplication, adaptation, distribution and reproduction in any medium or format, as long as you give appropriate credit to the original author(s) and the source, provide a link to the Creative Commons license, and indicate if changes were made.

The original article has been corrected.

Open Access This article is distributed under the terms of the Creative Commons Attribution 4.0 International License (http://creativecommons.org/licenses/by/4.0), which permits unrestricted use, distribution, and reproduction in any medium, provided you give appropriate credit to the original author(s) and the source, provide a link to the Creative Commons license, and indicate if changes were made. The images or other third party material in this article are included in the article’s Creative Commons licence, unless indicated otherwise in a credit line to the material. If material is not included in the article’s Creative Commons licence and your intended use is not permitted by statutory regulation or exceeds the permitted use, you will need to obtain permission directly from the copyright holder. To view a copy of this licence, visit http:// creativecommons.org/licenses/by/4.0/.

